# Clinical learning experiences and artificial intelligence–related anxiety among midwifery students: a cross-sectional study

**DOI:** 10.1186/s12909-026-09477-0

**Published:** 2026-06-03

**Authors:** Resmiye Özdilek, Eda Cangöl, Seda Cangöl Söğüt

**Affiliations:** 1 Faculty of Health Sciences, Division of Midwifery, Kocaeli University, Kocaeli, Türkiye; 2 Faculty of Health Sciences, Division of Midwifery, Canakkale Onsekiz Mart University, Canakkale, Türkiye

**Keywords:** Artificial intelligence, AI-related anxiety, Clinical learning environment, Midwifery education, Medical education

## Abstract

**Background:**

Artificial intelligence (AI) is rapidly transforming healthcare practice and education, requiring students to adapt to technology-supported clinical environments. Although AI-related anxiety has been examined among nursing students, little is known about how clinical learning experiences relate to AI-related anxiety among midwifery students. This study aimed to examine the relationship between clinical learning experiences and AI-related anxiety in undergraduate midwifery education.

**Methods:**

This cross-sectional study was conducted with 210 undergraduate midwifery students in Türkiye who were actively engaged in clinical training. Data were collected using validated measures assessing perceptions of the clinical learning environment and AI-related anxiety. Pearson correlation and simple linear regression analyses were performed to examine associations between clinical learning experiences and AI-related anxiety and its subdimensions.

**Results:**

Perceptions of the clinical learning environment were negatively associated with overall AI-related anxiety, job displacement anxiety, socio-technical blindness, and AI configuration anxiety (*p* < 0.05). No significant association was observed between clinical learning experiences and learning-related AI anxiety. Clinical learning experiences significantly predicted lower levels of overall AI-related anxiety.

**Conclusions:**

Clinical learning experiences appear to be associated with AI-related anxiety among midwifery students, particularly in relation to professional roles and system-level adaptation rather than learning difficulties. Strengthening clinical learning environments and integrating AI awareness into clinical education may support students’ preparedness for AI-enhanced healthcare practice.

**Supplementary Information:**

The online version contains supplementary material available at 10.1186/s12909-026-09477-0.

## Background

Health professions education is undergoing rapid transformation as digital technologies increasingly reshape clinical practice and professional competencies. Preparing students for technology-supported healthcare environments has therefore become a central educational challenge. Among these emerging technologies, artificial intelligence (AI) is gaining prominence not only as a clinical tool but also as a factor influencing students’ learning experiences, professional identity formation, and emotional adaptation within clinical education. Recent advances in artificial intelligence have accelerated the accessibility of AI-based applications, increasing their presence within both healthcare practice and health professions education [1].

As AI becomes increasingly integrated into healthcare systems, educational environments have also begun to adapt. Within educational contexts, AI technologies are reshaping teaching and learning processes by enabling adaptive, personalized, and data-driven learning experiences. Evidence suggests that such systems may enhance student engagement, promote self-directed learning, and support improved learning outcomes, particularly in professional education settings that require the integration of theoretical knowledge with clinical or practical skills [2]. Consequently, AI has become progressively embedded in educational environments, redefining pedagogical practices and students’ learning experiences.

However, the rapid digitalization of education has also introduced new challenges for students. Increased reliance on digital platforms, virtual learning environments, and technology-mediated instruction has shifted greater responsibility onto learners to manage complex technological systems. For many students, especially those enrolled in practice-based health professions, this transition may impose additional cognitive and emotional demands. Prior research indicates that intensive exposure to digital technologies may contribute to technostress and technology-related anxiety, which can adversely affect learning processes, psychological well-being, and academic performance [3].

Parallel to developments in education, AI technologies are increasingly integrated into healthcare systems, supporting clinical decision-making, risk assessment, documentation, predictive analytics, and patient monitoring [4, 5]. In nursing and midwifery practice, AI-based tools offer opportunities to enhance efficiency and quality of care while simultaneously reshaping professional roles and expectations. These changes require healthcare professionals to develop new competencies related to digital literacy, critical appraisal of AI outputs, and ethical decision-making.

Despite these potential benefits, the growing presence of AI in healthcare and educational contexts has raised concerns regarding professionals’ preparedness, role clarity, and emotional responses toward emerging technologies. Uncertainty about the reliability of AI systems, perceived threats to professional autonomy, and concerns about diminished professional competence may provoke anxiety toward AI, particularly among healthcare students who are still developing their professional identity and clinical confidence [6]. Such emotional responses may influence students’ engagement with AI-supported learning environments and their readiness to integrate AI into future clinical practice.

Clinical learning experiences constitute a core component of healthcare education, providing opportunities for experiential learning, interpersonal interaction, and the application of theoretical knowledge in real-world settings. For midwifery students, clinical education is especially central, as it emphasizes woman-centered care, continuity of care, professional judgment, and relational practice. The increasing integration of AI-supported systems into clinical environments may therefore intersect with midwifery students’ clinical learning experiences, shaping their perceptions of competence, autonomy, and professional role.

Recent research demonstrates a growing interest in the integration of AI into nursing and midwifery education, highlighting its potential to transform professional training and prepare students for technology-rich clinical environments. A comprehensive scoping review examining the predicted influence of AI-driven digital health technologies on nursing education emphasizes the urgent need for curricular reform and AI-informed pedagogical approaches in both academic and clinical settings [7]. AI-based tools, particularly simulation technologies and decision-support systems, have been shown to support clinical reasoning, preparedness for practice, and student engagement.

Nevertheless, emerging evidence suggests that the integration of AI into educational processes extends beyond technical and pedagogical considerations and directly affects students’ cognitive and emotional experiences. A recent scoping review synthesizing empirical studies on AI implementation in nursing education reports that AI-supported learning interventions may improve knowledge acquisition, engagement, and psychological safety, while simultaneously introducing challenges such as technical limitations, increased cognitive load, and heightened anxiety in some student populations [8]. These findings indicate that insufficient or poorly structured exposure to AI during professional training may contribute to uncertainty and artificial intelligence–related anxiety, potentially influencing students’ confidence, learning engagement, and clinical readiness.

From a theoretical perspective, artificial intelligence–related anxiety may arise from uncertainty about professional roles, perceived loss of control, and difficulties adapting to rapidly evolving technological environments. These concerns may be particularly relevant in healthcare education, where students are simultaneously developing clinical competence, professional identity, and confidence in patient care. Previous research suggests that technology-related anxiety is shaped not only by limited technical knowledge but also by perceptions of preparedness, self-efficacy, and the ability to maintain human-centered care within increasingly digital healthcare systems [2, 9]. In this context, supportive clinical learning experiences may help reduce uncertainty and strengthen students’ confidence in adapting to AI-integrated healthcare environments [10].

Despite the expanding role of AI in healthcare, empirical research examining healthcare students’ perceptions of AI—particularly in relation to their clinical learning experiences—remains limited. This gap is especially evident among midwifery students, whose education relies heavily on experiential learning, interpersonal engagement, and professional judgment. Compared with nursing students, midwifery students’ perceptions of AI and AI-related anxiety have received relatively little scholarly attention.

Accordingly, this study aims to examine the relationship between midwifery students’ clinical learning experiences and their levels of artificial intelligence–related anxiety. By addressing this gap, the study seeks to contribute evidence-based insights to the literature on AI in healthcare education and to inform curriculum development and educational strategies that support both technological competence and psychological well-being among midwifery students.

## Methods

### Study design and participants

This study employed a cross-sectional, descriptive, and correlational research design. Data were collected December 2025 through an online survey administered to undergraduate midwifery students in Turkey. The target population consisted of midwifery students who were actively engaged in clinical education during the data collection period.

Participants were recruited using a convenience sampling approach via online platforms. Eligible participants included second-, third-, and fourth-year undergraduate midwifery students who had prior clinical placement experience and voluntarily agreed to participate in the study. First-year students were excluded because they had not yet commenced clinical practice. Additional inclusion criteria were being aged 18 years or older and having access to a digital device capable of completing the online questionnaire. Students who did not provide informed consent or submitted incomplete questionnaires were excluded from the analysis.

The minimum required sample size was initially calculated using Epi Info™ version 7.2.5.0 based on a known population size (*N* = 312), a 5% margin of error, and a 90% confidence level, yielding a minimum sample size of 145 participants. Although the study aimed to reach the entire target population, participation was based on voluntary response and accessibility during the data collection period. To further evaluate the adequacy of the sample size for hypothesis testing, an a priori power analysis was conducted using GPower software (version 3.1.9.7). Based on a medium effect size (f² = 0.15), a significance level of α = 0.05, and a desired statistical power of 1–β = 0.95, the minimum required sample size was calculated as *N* = 107. The final sample consisted of 210 participants, exceeding both the minimum sample size estimates, and was therefore considered sufficient to conduct the planned statistical analyses. Data were collected using an anonymous, self-administered online questionnaire created via Google Forms. Prior to accessing the survey, participants were presented with an electronic informed consent form outlining the study purpose, procedures, voluntary participation, and data confidentiality. Only those who provided electronic consent were able to proceed with the questionnaire. Participation was entirely voluntary, and respondents were informed that they could withdraw from the study at any time without consequence. Ethical approval for the study was obtained from the Canakkale Onsekiz Mart University Health Sciences Ethics Committee (approval date: 8 December 2025). The study was conducted in accordance with the principles of the Declaration of Helsinki, and all data were collected anonymously to ensure participant confidentiality.

### Study hypotheses

Based on the study aim and the relevant literature, the following hypotheses were formulated to examine the relationship between midwifery students’ clinical learning experiences and artificial intelligence–related anxiety and its subdimensions:

- H1: Clinical learning experiences are negatively and significantly associated with the *learning-related anxiety* subdimension of artificial intelligence anxiety.
- H2: Clinical learning experiences are negatively and significantly associated with the *job displacement anxiety* subdimension of artificial intelligence anxiety.
- H3: Clinical learning experiences are negatively and significantly associated with the *socio-technical blindness* subdimension of artificial intelligence anxiety.
- H4: Clinical learning experiences are negatively and significantly associated with the *AI configuration anxiety* subdimension of artificial intelligence anxiety.
- H5: Clinical learning experiences are negatively and significantly associated with overall artificial intelligence–related anxiety.

### Instruments

#### Personal information form

A Personal Information Form consisting of 17 items was used to collect participants’ sociodemographic and educational characteristics. The form included questions related to age, gender, marital status, year of study, and other relevant background variables. These data were collected to describe the study sample and to support the interpretation of the study findings.

#### Midwifery students’ perceived clinical learning experiences scale

Midwifery students’ perceptions of their clinical learning experiences were assessed using the Midwifery Students’ Perceived Clinical Learning Experiences Scale (MSPCLES), developed by Griffiths et al. [11]. This scale was formed by combining the Clinical Learning Environment Scale and the Midwife Educator Influence Scale. Although both subscales can be used independently, their combined use has been reported to enhance measurement power.

The full scale consists of 26 items rated on a 4-point Likert scale (1 = *strongly disagree*, 4 = *strongly agree*), with total scores ranging from 26 to 104. The scale has no cutoff value; higher scores indicate more positive perceptions of clinical learning experiences. During the original scale development, content validity was established through expert evaluation, with an agreement rate of ≥ 83%. Exploratory factor analysis retained items with factor loadings ≥ 0.55, and internal consistency coefficients were reported as α = 0.92 for the Clinical Learning Environment subscale and α = 0.95 for the Midwife Educator Influence subscale [9].

In the present study, only the Clinical Learning Environment subdimension was used to assess students’ perceptions of their clinical learning experiences. This subdimension was selected because the study focused specifically on the relationship between clinical learning experiences and AI-related anxiety.

#### Artificial intelligence anxiety scale

Artificial intelligence–related anxiety was measured using the Artificial Intelligence Anxiety Scale (AIAS), a psychometric instrument developed by Wang and Wang [12] to assess individuals’ anxiety and perceptions regarding artificial intelligence technologies. The scale consists of four subdimensions: learning-related anxiety, job displacement anxiety, socio-technical blindness, and AI configuration anxiety.

The AIAS includes a total of 21 items rated on a 7-point Likert scale. The Turkish adaptation of the scale was conducted by Terzi (2020), and confirmatory factor analysis supported the validity of its four-factor structure in the Turkish sample. The scale demonstrated excellent internal consistency, with a reported overall Cronbach’s alpha coefficient of α = 0.96.

The Artificial Intelligence Anxiety Scale is a standardized data collection instrument that poses no risk to participants and is designed solely to assess individuals’ opinions and emotional responses toward artificial intelligence. The administration of the scale takes approximately 5–7 min. Participant responses were collected anonymously and used exclusively for scientific purposes [10].

### Data analysis

Data were analyzed using IBM SPSS Statistics version 26.0 (SPSS Inc., Chicago, IL, USA). The normality of the data distribution was evaluated using skewness and kurtosis values. All variables demonstrated skewness and kurtosis values within the acceptable range of ± 1.5, indicating approximate normal distribution [13]. (Table 3). Although the Kolmogorov–Smirnov test indicated statistically significant deviations from normality for all variables (*p* < 0.001), this result was interpreted with caution, as the test is known to be overly sensitive in large samples. Therefore, decisions regarding normality were based on skewness–kurtosis values and graphical inspections, in line with established recommendations [14].

Based on these assumptions, Pearson correlation analysis was conducted to examine the relationships between clinical learning experiences and artificial intelligence–related anxiety and its subdimensions. In addition, simple linear regression analysis was performed to assess the predictive effect of clinical learning experiences on artificial intelligence–related anxiety. The internal consistency of the measurement instruments was evaluated using Cronbach’s alpha coefficient, with values indicating acceptable reliability.

## Results

### Sociodemographic characteristics of participants

A total of 210 midwifery students participated in the study. The mean age of the participants was 21.00 ± 2.25 years (range: 18–42). The mean grade point average was 3.07 ± 0.33, ranging from 1.80 to 3.87. The majority of participants were single (97.6%, *n* = 205), and 44.7% (*n* = 94) reported living in households with four or more individuals.

Regarding academic year, 34.3% (*n* = 72) were fourth-year students, while 33.3% (*n* = 70) and 32.4% (*n* = 68) were in the second and third years, respectively. Most participants reported residing in urban areas (48.1%, *n* = 101). Following graduation, 71.9% (*n* = 151) indicated a preference for working in public hospitals. Detailed sociodemographic characteristics of the participants are presented in Table 1.

**Table 1:** Sociodemographic characteristics of participants (*N* = 210)

Age* (21.00 ± 2.25; min-max:18–42)		
Grade Point Average (3.07 ± 0.33; min-max:1.80–3.87)		
	*N*	%
Age		
Age 18–24	206	98.1
Age ≥ 25	4	1.9
Marital status		
Single	205	97.6
Married	5	2.4
Year of study		
2	70	33.3
3	68	32.4
4	72	34.3
Place of residence		
Rural area	28	13.3
District	81	38.6
City center	101	48.1
Mother’s education level		
Illiterate	9	4.3
Literate	11	5.2
Primary school	89	42.4
Secondary school	39	18.6
High school	55	26.2
University or higher	7	3.3
Father’s education level		
Illiterate	3	1.4
Literate	1	0.5
Primary school	64	30.5
Secondary school	41	19.5
High school	75	35.7
University or higher	26	12.4
Number of household members		
2	7	3.3
3	25	11.9
4	84	40.1
4 +	94	44.7
Employment status		
Not working	187	89.0
Part-time	23	11.0
Perceived economic status		
Low	41	19.5
Moderate	166	79.0
High	3	1.4
Preferred workplace after graduation		
Public hospital	151	71.9
Private hospital	4	1.9
Family health center	26	12.4
Abroad	17	8.1
Other	6	5.7

^*^Mean ± SD

### Technology-related characteristics

Most participants (90.0%, *n* = 189) reported that they had not previously received any education or training related to artificial intelligence. Regarding AI usage experience, 44.3% (*n* = 93) indicated having limited experience, while only 1.9% (*n* = 4) reported advanced experience. The majority of participants (71.0%, *n* = 149) rated their computer and technology competence as moderate.

In terms of technology use frequency, 66.2% (*n* = 139) reported using computers on a daily basis. Half of the participants (50.0%, *n* = 105) described their educational program as balanced between theoretical and clinical components. Technology-related characteristics are summarized in Table 2.

**Table 2:** Participants’ technology and artificial intelligence–related experiences (*N* = 210)

	*N*	%
Previous AI-related training		
Yes	21	10.0
No	189	90.0
AI usage experience		
None	32	15.2
Low	93	44.3
Moderate	81	38.6
Advanced	4	1.9
Computer and technology competence		
Very low	10	4.8
Low	36	17.1
Moderate	149	71.0
High	14	6.6
Very high	1	0.5
Family/environmental support for technology use		
Insufficient	84	40.0
Partially sufficient	98	46.7
Sufficient	28	13.3
Frequency of computer use		
Daily	139	66.2
Several times per week	34	16.2
Several times per month	13	6.2
Rarely	24	11.4
Structure of the curriculum		
Predominantly theoretical	50	23.8
Predominantly clinical	55	26.2
Balanced	105	50.0

### Descriptive statistics and reliability of measurement instruments

Descriptive statistics and internal consistency coefficients for the study variables are presented in Table 3. The Clinical Learning Environment subscale demonstrated excellent internal consistency (Cronbach’s α = 0.956), with a mean score of 44.1 ± 8.89. The Artificial Intelligence Anxiety Scale also showed high internal consistency (α = 0.949), with a mean total score of 79.5 ± 24.8.

Cronbach’s alpha coefficients for the AI anxiety subdimensions were 0.927 for learning-related anxiety, 0.947 for job displacement anxiety, 0.909 for socio-technical blindness, and 0.931 for AI configuration anxiety. Skewness and kurtosis values for all variables were within acceptable ranges, supporting the assumption of normality (Table 3).

**Table 3:** Descriptive statistics of measurement instruments

Scale / Subscale	Cronbach’s α	Mean	SD	Skewness	Kurtosis	Min	Max
MSPCLES	0.956	44.1	8.89	-0.31	0.91	16	64
AIAS- Learning-related	0.927	23.2	9.7	-0.23	-0.05	8	56
AIAS- Job displacement	0.947	26	9.51	0.39	0.13	6	42
AIAS- Socio-technical blindness	0.909	17.5	6.28	-0.22	-0.63	4	28
AIAS- AI configuration	0.931	12.8	5.15	-0.03	-0.79	3	21
AIAS- Total	0.949	79.5	24.8	0.06	-0.84	21	147

X̄: Mean

### Correlations between clinical learning experiences and artificial intelligence anxiety

Pearson correlation analysis revealed no statistically significant relationship between clinical learning experiences and learning-related anxiety (*p* > 0.05). However, clinical learning experiences were negatively and significantly correlated with job displacement anxiety (*r* = − 0.190, *p* < 0.05), socio-technical blindness (*r* = − 0.243, *p* < 0.05), AI configuration anxiety (*r* = − 0.194, *p* < 0.05), and overall artificial intelligence anxiety (*r* = − 0.166, *p* < 0.05). Correlation coefficients among all study variables are presented in Table 4.

**Table 4:** Results of pearson correlation analysis

Scale / Subscale	1	2	3	4	5	6
1. MSPCLES	1	-0.022	-0.190^**^	-0.243^**^	-0.194^**^	-0.166^**^
2. AIAS- Learning-related		1	0.389^*^	0.324^**^	0.327^*^	0.690^*^
3. AIAS- Job displacement			1	0.826^*^	0.724^*^	0.895^*^
4. AIAS- Socio-technical blindness				1	0.791^*^	0.861^*^
5. AIAS- AI configuration					1	0.814^**^
6. AIAS- Total						1

^*^p < 0.0001; ^**^p < 0.05

### Regression analysis

A simple linear regression analysis was conducted to examine whether clinical learning experiences predicted artificial intelligence–related anxiety. The regression model was statistically significant (*F* = 5.92, *p* < 0.05). Clinical learning experiences were found to be a significant negative predictor of artificial intelligence anxiety (β = −0.16, *p* < 0.05; 95% CI [− 0.30, − 0.03]). The model explained 2.7% of the variance in artificial intelligence anxiety (*R²* = 0.027). Detailed regression results are presented in Table 5.

**Table 5:** Results of regression analysis between MSPCLES and AIAS

Independent Variable	B	SE	β	t	*p*	95% CI	
						Lower	Upper
Constant	99.92	8.57		11.66	**< 0.001**	41.81	55.50
MSPCLES	-0.464	0.19	-0.16	-2.43	**0.016**	-0.30	-0.03

R² = 0.027, F(1,209) = 5.92

Bold values indicate statistical significance at *p* < .05.

## Discussion

In this study, significant relationships were identified between midwifery students’ perceptions of their clinical learning experiences and their levels of artificial intelligence (AI)–related anxiety. Students who perceived the clinical learning environment more positively reported lower levels of overall AI anxiety, as well as lower levels of job displacement anxiety, socio-technical blindness, and AI configuration anxiety. These findings indicate that clinical learning environments shape not only professional skill development but also students’ emotional and cognitive responses toward emerging technologies.

Although the regression model was statistically significant, the relatively low explained variance indicates that clinical learning experiences may represent only one aspect of AI-related anxiety among midwifery students. AI-related anxiety is likely shaped by multiple factors, including digital literacy, prior experience with technology, academic stress, coping styles, and perceptions of professional preparedness. Therefore, the findings should be interpreted within the broader multidimensional nature of students’ emotional responses to AI-supported healthcare environments.

Previous research supports the role of the clinical learning environment in buffering students’ stress and anxiety. Mazalová et al. (2022) demonstrated that supportive supervision, positive instructor–student relationships, and a structured pedagogical climate were associated with lower levels of academic and clinical stress among nursing students. Within this framework, high-quality clinical learning experiences may reduce students’ perceptions of threat when encountering complex and unfamiliar technologies, including AI [15].

Consistent with this interpretation, readiness for AI has been identified as a key determinant of AI-related anxiety among midwifery and nursing students, particularly in relation to perceived threats to professional roles [16]. In the present study, students who reported more positive clinical learning experiences also demonstrated lower levels of job displacement anxiety. Technology-integrated clinical learning environments may help reduce anxiety by enhancing confidence during clinical decision-making [17].

Prior studies indicate that healthcare students often demonstrate moderate levels of AI literacy and self-efficacy, which may buffer learning-related anxiety [18]. In line with previous findings, learning-related concerns appear to be less salient than anxieties related to professional roles and system-level implications, underscoring the multidimensional nature of AI anxiety [19]. The significant negative association between clinical learning experiences and job displacement anxiety further highlights the importance of professional identity formation. Clinical environments that promote active participation, responsibility, and skill acquisition may reinforce students’ confidence in the human-centered dimensions of midwifery practice, such as relational care, clinical judgment, and contextual decision-making. Supporting this view, AI-related job anxiety has been shown to intensify when healthcare professionals perceive a loss of control and accountability in clinical processes [20]. For midwifery students, whose education emphasizes woman-centered and relational care, AI is therefore less likely to be perceived as a replacement when students feel professionally grounded.

The strong negative association between clinical learning experiences and socio-technical blindness represents one of the most important contributions of this study. As emphasized by McCradden et al. (2023), AI should be conceptualized as a socio-technical system embedded within institutional practices, ethical frameworks, and human decision-making processes rather than as an isolated technical tool. From this perspective, socio-technical blindness reflects limited awareness of how AI systems operate within broader clinical and organizational structures [21]. Consistent with this interpretation, readiness for AI has been identified as a key determinant of AI-related anxiety among midwifery and nursing students, particularly in relation to perceived threats to professional roles [9]. In the present study, students who reported more positive clinical learning experiences also demonstrated lower levels of job displacement anxiety.

No significant relationship was found between perceptions of the clinical learning environment and learning-related AI anxiety. This finding suggests that AI-related anxiety among midwifery students may be driven less by concerns about learning new technologies and more by uncertainties related to professional roles and system-level implications. Previous studies also indicate that healthcare students often report moderate levels of AI literacy and self-efficacy, which may buffer learning-related anxiety [18, 19]. In contrast, the negative association between clinical learning experiences and job displacement anxiety highlights the importance of professional identity formation. Clinical environments that promote responsibility, participation, and skill development may strengthen students’ confidence in the human-centered aspects of midwifery care and reduce perceived threats related to AI-driven role replacement [20].

Similarly, the negative association between clinical learning experiences and AI configuration anxiety suggests that students’ concerns stem less from AI itself and more from perceived exclusion from decision-making processes. Nurse educators play a critical role in modelling ethical reasoning, transparency, and boundary-setting in relation to AI systems [22].

Moreover, limited transparency, accountability, and participatory governance in AI integration have been identified as key contributors to anxiety and resistance among healthcare students and professionals [23]. Supportive clinical learning environments may therefore reduce AI configuration anxiety by enabling students to recognize AI systems as socially constructed, ethically guided, and professionally influenceable rather than fixed and uncontrollable technologies.

Taken together, these findings indicate that reducing AI-related anxiety in midwifery education requires more than increased technical exposure to AI tools. Instead, AI education should be embedded within clinical learning experiences that foster professional confidence, socio-technical awareness, and agency. From this perspective, mitigating AI anxiety involves positioning students as active participants within socio-technical systems rather than passive recipients of technological change.

### Strengths and limitations

This study is among the few empirical investigations examining artificial intelligence–related anxiety among midwifery students in relation to their clinical learning experiences. A key strength lies in the examination of multiple AI anxiety subdimensions, providing a nuanced understanding of students’ concerns beyond general technology-related anxiety. The use of validated and reliable measurement instruments further strengthens the robustness of the findings.

However, the cross-sectional design limits causal interpretations, and the reliance on self-reported data may introduce response bias. In addition, the use of convenience sampling within a single national context may restrict the generalizability of the results. Future studies employing longitudinal designs and more diverse samples are recommended to extend these findings. Furthermore, online data collection and convenience sampling may have introduced selection bias related to technology access and interest in AI-related topics.

## Conclusions

This study demonstrates that midwifery students’ perceptions of their clinical learning environment are meaningfully associated with their levels of artificial intelligence–related anxiety. Positive clinical learning experiences were linked to lower levels of job displacement anxiety, socio-technical blindness, AI configuration anxiety, and overall AI anxiety, while no significant association was observed with learning-related anxiety. These findings suggest that AI-related concerns among midwifery students are shaped less by difficulties in learning new technologies and more by uncertainties related to professional roles, system-level integration, and control. Embedding AI awareness within supportive clinical learning environments may therefore play a key role in fostering socio-technical understanding and professional confidence.

From an educational perspective, integrating AI literacy, ethical AI use, and critical reflection on technology-supported care into midwifery curricula may help students feel more prepared and confident in AI-enhanced clinical environments. Clinical educators may also play an important role in supporting students’ adaptation to AI-integrated healthcare systems through guidance, discussion, and reflective learning opportunities. Addressing AI-related anxiety through clinically grounded educational strategies may support midwifery students’ readiness to engage with AI-enhanced healthcare practices.

## Supplementary Information

Supplementary Material 1.

## Data Availability

All data and materials in this article are included in the manuscript. The raw data could be obtained from corresponding author upon a reasonable request.
