# Supplementary material for: Clinical learning experiences and artificial intelligence–related anxiety among midwifery students: a cross-sectional study
Source: BMC Med Educ. 2026 Jun 3;26:1201. doi: 10.1186/s12909-026-09477-0 (PMC13393852; doi:10.1186/s12909-026-09477-0)
Supplement: Supplementary file 1 — Supplementary Material 1. [file 12909_2026_9477_MOESM1_ESM.docx]

STROBE Statement—checklist of items that should be included in reports of observational studies

|  | Item No. | Recommendation | Page  No. | Relevant text from manuscript |
| --- | --- | --- | --- | --- |
| **Title and abstract** | 1 | (*a*) Indicate the study’s design with a commonly used term in the title or the abstract | 1 | The study design (cross-sectional) is stated in the abstract. |
|  |  | (*b*) Provide in the abstract an informative and balanced summary of what was done and what was found | 1 | The abstract provides a structured and balanced summary of the study background,  methods, results, and conclusions. |
| Introduction | | | |  |
| Background/rationale | 2 | Explain the scientific background and rationale for the investigation being reported | 2-3 | The introduction explains the scientific background and rationale for examining artificial intelligence–related anxiety in relation to clinical learning experiences among midwifery students. |
| Objectives | 3 | State specific objectives, including any prespecified hypotheses | 3 | The objectives and hypotheses of the study are clearly stated. |
| Methods | | | |  |
| Study design | 4 | Present key elements of study design early in the paper | 4 | A cross-sectional, descriptive, and correlational study design  is described. |
| Setting | 5 | Describe the setting, locations, and relevant dates, including periods of recruitment, exposure, follow-up, and data collection | 4 | The study setting, location, and data collection period  are reported. |
| Participants | 6 | (*a*) *Cohort study*—Give the eligibility criteria, and the sources and methods of selection of participants. Describe methods of follow-up  *Case-control study*—Give the eligibility criteria, and the sources and methods of case ascertainment and control selection. Give the rationale for the choice of cases and controls  *Cross-sectional study*—Give the eligibility criteria, and the sources and methods of selection of participants | 4-5 | Eligibility criteria and the sources and methods of participant  selection are described for this cross-sectional study. |
|  |  | (*b*) *Cohort study*—For matched studies, give matching criteria and number of exposed and unexposed  *Case-control study*—For matched studies, give matching criteria and the number of controls per case |  | - |
| Variables | 7 | Clearly define all outcomes, exposures, predictors, potential confounders, and effect modifiers. Give diagnostic criteria, if applicable | 5 | All outcome variables, predictors, and measurement instruments  used in the study are clearly defined. |
| Data sources/ measurement | 8* | For each variable of interest, give sources of data and details of methods of assessment (measurement). Describe comparability of assessment methods if there is more than one group | 5-6 | Data sources and methods of assessment are described using  validated and reliable measurement instruments. |
| Bias | 9 | Describe any efforts to address potential sources of bias | 6 | Potential sources of bias and strategies to minimize bias  are addressed. |
| Study size | 10 | Explain how the study size was arrived at | 6 | The study size was determined based on sample size calculation  and power analysis. |

Continued on next page

| Quantitative variables | 11 | Explain how quantitative variables were handled in the analyses. If applicable, describe which groupings were chosen and why | 6 | Quantitative variables were handled using appropriate statistical  methods, and no categorization or grouping was applied. |
| --- | --- | --- | --- | --- |
| Statistical methods | 12 | (*a*) Describe all statistical methods, including those used to control for confounding | 6-7 | All statistical methods used in the analysis, including correlation  and regression analyses, are described. |
|  |  | (*b*) Describe any methods used to examine subgroups and interactions | - | No subgroup or interaction analyses were conducted. |
|  |  | (*c*) Explain how missing data were addressed | 7 | Missing data were minimal and handled according to standard  statistical procedures. |
|  |  | (*d*) *Cohort study*—If applicable, explain how loss to follow-up was addressed  *Case-control study*—If applicable, explain how matching of cases and controls was addressed  *Cross-sectional study*—If applicable, describe analytical methods taking account of sampling strategy | - | Not applicable for this cross-sectional study. |
|  |  | (*e*) Describe any sensitivity analyses | - | No sensitivity analyses were performed. |
| Results | | | | |
| Participants | 13* | (a) Report numbers of individuals at each stage of study—eg numbers potentially eligible, examined for eligibility, confirmed eligible, included in the study, completing follow-up, and analysed | 7 | The number of participants included in the study and analyzed  is reported. |
|  |  | (b) Give reasons for non-participation at each stage | - | Reasons for non-participation were not applicable. |
|  |  | (c) Consider use of a flow diagram | - | A flow diagram was not required for this study. |
| Descriptive data | 14* | (a) Give characteristics of study participants (eg demographic, clinical, social) and information on exposures and potential confounders | 7-8 | Demographic and descriptive characteristics of the study  participants are presented. |
|  |  | (b) Indicate number of participants with missing data for each variable of interest | - | The number of participants with missing data was minimal  and is reported. |
|  |  | (c) *Cohort study*—Summarise follow-up time (eg, average and total amount) | - | Not applicable for this cross-sectional study. |
| Outcome data | 15* | *Cohort study*—Report numbers of outcome events or summary measures over time |  |  |
|  |  | *Case-control study—*Report numbers in each exposure category, or summary measures of exposure |  |  |
|  |  | *Cross-sectional study—*Report numbers of outcome events or summary measures | 8 | Summary measures of the outcome variables are reported  for this cross-sectional study. |
| Main results | 16 | (*a*) Give unadjusted estimates and, if applicable, confounder-adjusted estimates and their precision (eg, 95% confidence interval). Make clear which confounders were adjusted for and why they were included | 8-9 | Main results, including correlation and regression analyses  with corresponding significance levels, are reported. |
|  |  | (*b*) Report category boundaries when continuous variables were categorized | - | Continuous variables were not categorized. |
|  |  | (*c*) If relevant, consider translating estimates of relative risk into absolute risk for a meaningful time period | - | Not applicable for this cross-sectional study. |

Continued on next page

| Other analyses | 17 | Report other analyses done—eg analyses of subgroups and interactions, and sensitivity analyses |  | No additional analyses, including subgroup or sensitivity analyses,  were performed. |
| --- | --- | --- | --- | --- |
| Discussion | | | | |
| Key results | 18 | Summarise key results with reference to study objectives | 9 | Key findings are summarized in relation to the study objectives  in the discussion section. |
| Limitations | 19 | Discuss limitations of the study, taking into account sources of potential bias or imprecision. Discuss both direction and magnitude of any potential bias | 10 | Study limitations and potential sources of bias are discussed. |
| Interpretation | 20 | Give a cautious overall interpretation of results considering objectives, limitations, multiplicity of analyses, results from similar studies, and other relevant evidence | 9-10 | The results are interpreted cautiously in light of the study  objectives, limitations, and existing literature. |
| Generalisability | 21 | Discuss the generalisability (external validity) of the study results | 10 | The generalisability of the study findings is discussed. |
| Other information | |  | | |
| Funding | 22 | Give the source of funding and the role of the funders for the present study and, if applicable, for the original study on which the present article is based | 10 | The study received no external funding. |

*Give information separately for cases and controls in case-control studies and, if applicable, for exposed and unexposed groups in cohort and cross-sectional studies.

**Note:** An Explanation and Elaboration article discusses each checklist item and gives methodological background and published examples of transparent reporting. The STROBE checklist is best used in conjunction with this article (freely available on the Web sites of PLoS Medicine at http://www.plosmedicine.org/, Annals of Internal Medicine at http://www.annals.org/, and Epidemiology at http://www.epidem.com/). Information on the STROBE Initiative is available at www.strobe-statement.org.
